# Population-based estimates of age and comorbidity specific life expectancy: a first application in Swedish males

**DOI:** 10.1186/s12911-022-01766-0

**Published:** 2022-02-08

**Authors:** Mieke Van Hemelrijck, Eugenio Ventimiglia, David Robinson, Rolf Gedeborg, Lars Holmberg, Pär Stattin, Hans Garmo

**Affiliations:** 1 Translational Oncology & Urology Research (TOUR), School of Cancer and Pharmaceutical Sciences, Guy’s Hospital, King’s College London, 3rd flr Bermondsey Wing, London, SE1 9RT UK; 2 Regional Cancercenter Mellansverige, Regional Cancercenter Mellansverige, Uppsala, Sweden; 3 Department of Surgical Sciences, Uppsala University, Uppsala, Sweden; 4 Department of Urology, Ryhov Hospital, Jönköping, Sweden; 5 Division of Experimental Oncology/Unit of Urology, IRCCS Ospedale San Raffaele, Milan, Italy

**Keywords:** Charlson Comorbidity Index, Life expectancy, State transition models, Prostate cancer

## Abstract

**Introduction:**

For clinical decision-making, an estimate of remaining lifetime is needed to assess benefit against harm of a treatment during the remaining lifespan. Here, we describe how to predict life expectancy based on age, Charlson Comorbidity Index (CCI) and a Drug Comorbidity Index (DCI), whilst also considering potential future changes in CCI and DCI using population-based data on Swedish men.

**Methods:**

Simulations based on annual updates of vital status, CCI and DCI were used to estimate life expectancy at population level. The probabilities of these transitions were determined from generalised linear models using prostate cancer-free comparison men in PCBaSe Sweden. A simulation was performed for each combination of age, CCI, and DCI. Survival curves were created and compared to observed survival. Life expectancy was then calculated as the area under the simulated survival curve.

**Results:**

There was good agreement between observed and simulated survival curves for most ages and comorbidities, except for younger men. With increasing age and comorbidity, there was a decrease in life expectancy. Cross-validation based on six regions in Sweden also showed that simulated and observed survival was similar.

**Conclusion:**

Our proposed method provides an alternative statistical approach to estimate life expectancy at population level based on age and comorbidity assessed by routinely collected information on diagnoses and filled prescriptions available in nationwide health care registers.

## Introduction

For clinical decision-making, an estimate of remaining lifetime is needed to weigh the benefit of a specific treatment against its the potential harm during the remaining lifespan. However, most life tables models available today do not take comorbidity into account [1], despite the fact that comorbidity strongly affects life expectancy [2, 3]. More recently, some new approaches have been presented to calculate comorbidity-adjusted life expectancy [2, 4, 5]. Nevertheless, this methodology is usually based on the use of flexible parametric or semi-parametric models or a static baseline assessment of comorbidity levels [4, 6], and may overestimate life expectancy as these models can only be applied to periods for which data is available, without taking into account comorbidity changes during follow-up [1].

Here, we present an alternative statistical model developed in a male cohort using age and measurements of comorbidity based on hospital discharge diagnoses as well as filled drug prescriptions collected at a population-based level to estimate life expectancy. We propose to use life tables for each level of age and comorbidity and allow this to change during follow-up by use of a state transition model [7].

## Methods

To assess the risk of death and changes in comorbidity, we used the comparison cohort of Prostate Cancer data Base Sweden (PCBaSe) 4.0, a cohort of men without prostate cancer who were matched with men with prostate cancer based on birth year and county of residence [8]. We included without prostate cancer as to account for health-seeking behaviour (i.e. many men get diagnosed with localised PCa due to opportunistic screening and are therefore often healthier than the general population [9]). All men aged 65–90 years at entrance to the cohort between 1 Jan 2007 to 31 December 2013 were included (N = 230,223). Follow-up ended at date of death, date of emigration, or 21 December 2017, whichever occurred first.

Charlson Comorbidity Index (CC) [10] was calculated based on data on discharge diagnoses in the National Patient Registry [11]. Similarly, a Drug Comorbidity Index (DCI) [3, 12] was calculated based on data on filled prescriptions in the Prescribed Drug Registry [11]. Both the CCI and DCI were calculated at date of entry to the cohort and in each consecutive year until end of follow-up. For CCI, we used a cumulative CCI including all events dating back to 10 years prior to entry to the cohort. For the DCI, we used prescriptions filled in the previous year.

Our method thus predicts life expectancy at a population-level based on current age, CCI and DCI, whilst taking into account future changes in comorbidity as a dynamic process, using population-based data on Swedish men as a first application.

### Simulation algorithm to estimate life expectancy

Life expectancy was calculated based on the output from a state transition model microsimulation. The algorithm applied to each man in the microsimulation involved the following steps:

1. Has the man already died according to the simulation? If yes, exit the update process. If no, continue to step 2.
2. Will the man die in the next year according to the simulation? If yes, record the death and exit the update process. If no, continue to step 3.
3. Will a change in CCI occur during the next year according to the simulation? If no, record the old CCI and go to step 5. If yes, continue to step 4.
4. Determine the size of CCI change according to Lindhagen et al. [13] and record the new CCI.
5. Will a change in DCI occur during the next year according to the simulation? If no, record the old DCI and exit the update process. If yes, continue to step 6.
6. Will the DCI increase according to the prediction? If yes, determine the size of increase and record the new DCI and exit the update process. Otherwise go to step 7.
7. Determine the size of decrease and record the new DCI.
8. Increase age by one and go to step 1 and repeat until all men are dead or of age = 105.

Note that the algorithm will give the same output if steps 5–7 are performed prior to steps 3–4.

The algorithm was implemented in R [14] and the simulation code is presented in the “Appendix”.

### Estimation of model parameters in the state transition model

The comparison cohort of PCBaSe 4.0 was used to estimate model parameters. The follow-up of the men in our cohort was transformed into long format, i.e. one row of data with updated age, CCI and DCI for each year of follow-up (Table 1 in “Appendix”). Probabilities of state transitions in 1 year were determined from generalised linear models as described below—the parameters from these models were then used for the micro-simulation (see below). Logistic regression was used for modelling dichotomised events such as death (yes/no), any change in CCI (yes/no), and any change in DCI (yes/no,) whereas size of changes was modelled through Poisson- and Gamma regression (Table 2 in “Appendix”).

#### Modelling of the probability of death

For the probability of death, we used a standard life table modelling approach [15]. First, we noted that CCI and DCI were associated with death in a non-linear manner since an increase from zero to one unit affected the risk of death more strongly than a one-unit change in higher levels CCI and DCI. Therefore, we used a function that was constant for all x-values above a specific value (cut point) and joined to a half parabola to the left of the cut point, i.e., a second-degree polynomial with its maximum/minimum in the cut point (Table 2). We further described this quadratic-constant spline (QCS) as follows. For CCI, we used the cut-point seven in the QCS, whereas for DCI we used 14 as the cut-point. Moreover, the effect of CCI and DCI on death was decreasing with age and therefore we modelled the interaction between age and CCI/DCI as using a QCS with cut-point 100 years. Details regarding the model are presented in Table 2.

#### Modelling of CCI

Changes in CCI were modelled as previously described [13]. This method used a two-step procedure: (1) assessment of whether a change in CCI occurred; (2) determination of the size of change if this indeed occurred. The last step was implemented using two Poisson regression models. In these models we categorised both CCI and DCI. Details of the models and categorisation are shown in Tables 1 and 2.

#### Modelling of DCI

To model changes in DCI, we modified the method presented by Lindhagen et al. [13]. We used a four-step procedure: (1) assessment of whether a change in DCI occurred and if so whether this was an increase or decrease; (2) determination of the size of change if this indeed occurred (either decrease or increase). The size of DCI change was determined using a generalised linear Gamma model with a log link. Details of the models and categorisation are shown in Tables 1 and 2.

#### Microsimulation

The above steps yielded a set of parameter estimates which were used to simulate death, CCI and DCI in a microsimulation [9], i.e., a simulation of changes in vital status, CCI, and DCI for individual study subjects. In this simulation, outcomes according to the simulation algorithm above were generated. From the simulation, the proportion of deaths in each time step was calculated and used to create a survival curve. For each combination of age (65, 66, …, 90), CCI (0, 1, 2, …, 10), and DCI (− 0.75, − 0.5, …, 13.5), we ran the microsimulation using 10,000 identical men. The life expectancy was calculated as the area under the survival curve emanating from the simulation. No man was considered alive beyond the age of 105.

#### Validation

A validation where observed and simulated data is compared was impossible in this setting as the expected remaining lifetime corresponds to the area under survival curve that drops to zero. For some combinations of age, CCC and DCI this would require a follow-up of more than 40 years. Our data allows for maximal possible follow-up of 11 years. Therefore, the validation relies on a set of comparisons which indirectly served as a validation. First, we compared simulated and observed survival for men based on age, CCI and DCI based on 11 years of follow up. Next, we compared the observed change in mean CCI and DCI over time following cohort entry and the corresponding change in simulated mean CCI To further validate our simulation model we created calibration plots for the death model (Fig. 9) and for the models used to capture DCI changes (Fig. 10). Finally, we assessed the validity of our method in a cross-validation by splitting our comparison cohort of PCBaSe 4.0 based on the six health care regions in Sweden. For each health care region, data from men in the other five regions were used to estimate transition probabilities.

## Results

**Fig. 1:**
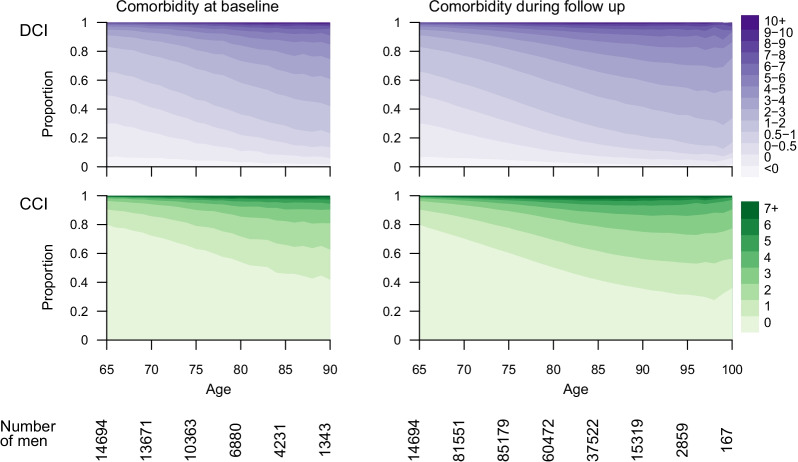
Charlson Comorbidity Index (CCI) and Drug Comorbidity Index (DCI) for men in the comparison cohort of prostate cancer-free men in Prostate Cancer data Base 4.0 according to age at entry to cohort and during follow-up

In our dataset, CCI increased with age. Around 80% of men aged 65 at entry to the cohort had CCI = 0, whilst among men aged 90 the prevalence of CCI = 0 was 45% (Fig. 1). Similarly, DCI also increased with age. For men aged 65 at entry to the cohort, the prevalence of DCI > 0 was 70% and the corresponding number in men aged 90 was 85%. Similar increases in CCI and DCI were observed during increasingly long follow-up (Fig. 1).

**Fig. 2:**
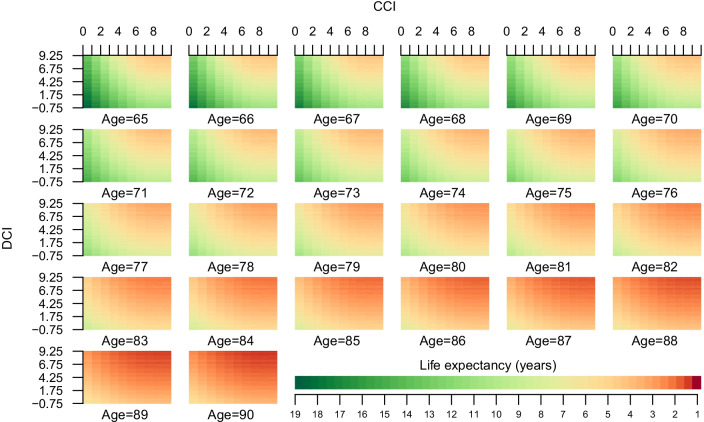
Life expectancy based on age, Charlson Comorbidity Index and Drug Comorbidity Index

**Fig. 3:**
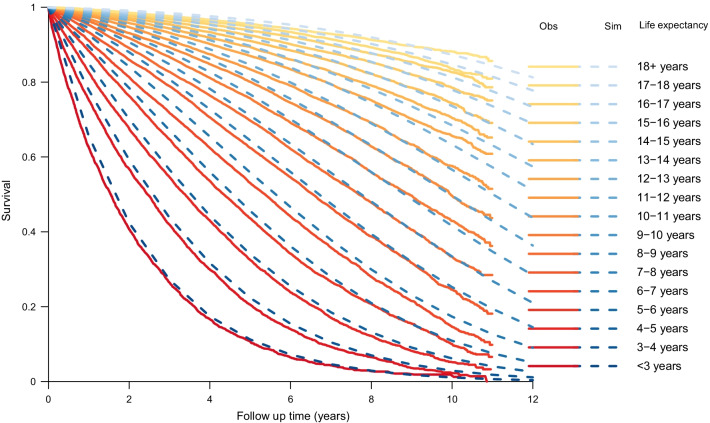
Simulated and observed survival for men by life expectancy

For each age, there was a decrease in life expectancy for a fixed CCI, when increasing the DCI and for a fixed DCI, when increasing the CCI (Fig. 2). When splitting the estimated lifetime expectancy into 1-year categories and comparing simulated survival curves and observed curves, the predicted survival in the simulated curves was somewhat greater than in observed curves during the first 5 years, but at 10 years the observed and simulated curves where almost similar (Fig. 3). The years lost, i.e., the area above the survival curve, during the first 10 years of follow up was similar between observed and simulated survival curves and differed at most 3 months for the group with expected remaining lifetime 6–7 years (Table 3).

**Fig. 4:**
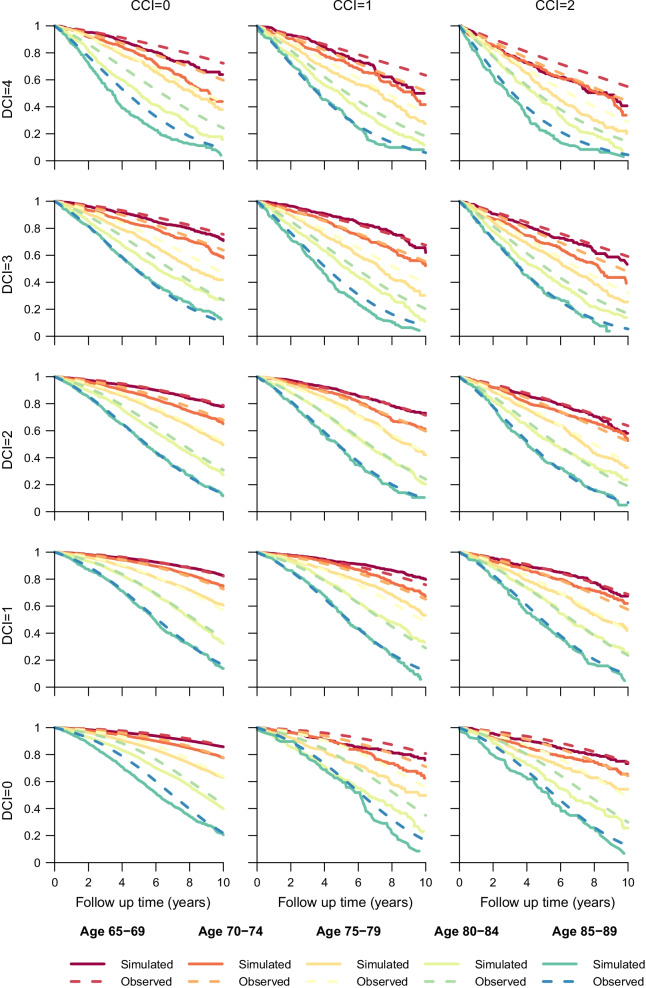
Simulated and observed survival for men based on age at entrance to the cohort, and CCI and DCI status

As part of our validation, we compared simulated and observed survival for men based on age, CCI and DCI (Fig. 4). No obvious systematic differences were found. Upon comparing the observed change in mean CCI and DCI over time following cohort entry and the corresponding change in simulated mean CCI (Figs. 6 and 7 in “Appendix”), results were found to be similar for most CCI strata, but for DCI = 3 and 4 we noted a difference between observed and simulated from year 2 onwards.

**Fig. 5:**
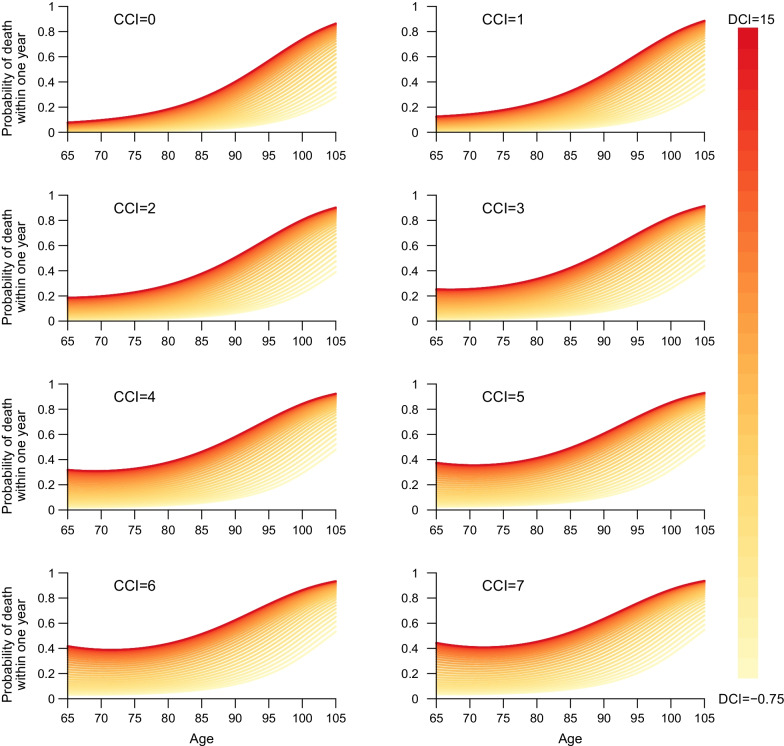
Modelled probability of death within 1 year based on levels of CCI, DCI, and age

**Fig. 6:**
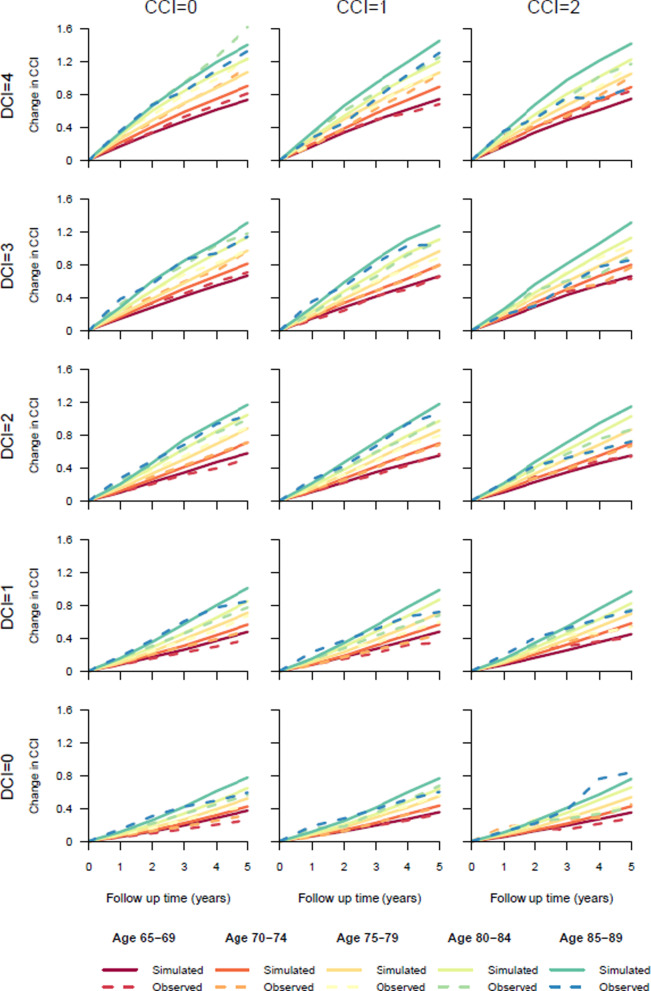
Estimated and observed mean change in CCI based on DCI and age

The probability of death within the next year, corresponding to the applied life tables was found to be increasing with age, CCI and DCI (Figs. 5, 11). The effect of increasing DCI for a fixed CCI was increasing with age. For higher levels of CCI the relation between DCI and age was attenuated. The predicted mortality risk based on age, CCI, and DCI corresponded well to the observed risk (Fig. 8). The calibration plots for our DCI change modelling approach showed good agreement between predicted and observed probability of DCI-change (Fig. 9, panel a). Similar agreement was found for the probability of an increase (Fig. 9, panel b). The size of DCI-change was somewhat underestimated (Fig. 9, panels c and d).

Finally, our cross-validation based on the six health care regions in Sweden showed that simulated and observed survival was similar for all regions in each cross-validation (Fig. 8, Table 4).

## Discussion

Life expectancy at a population level was quantified based on age and changes in comorbidity based on the Charlson Comorbidity Index and a new Drug Comorbidity Index. The observed and simulated survival curves were similar up to 9 years of follow-up for men with higher age, CCI and DCI. The models accurately predicted changes in CCI and DCI.

There are several methods that measure comorbidity [16]. CCI is the most commonly used measurement for comorbidity status [17] and was originally based on 17 medical conditions and their severity with the aim to predicted 1-year mortality. CCI has been modified for use with administrative data such as ICD 9 and ICD 10 coding in health care registries and is usually applied to discharge diagnoses. Similarly, we have created a prescription-based comorbidity index based on fillings in a Prescribed Drug Registry to complement CCI [3, 12]. The advantages of our DCI is that it adds predictive ability beyond what the CCI already provides [3]. Measurements of comorbidity are of limited use for long-term predictions since they were created to predict the risk of death typically from 1 to 3 years. A preferred alternative is therefore based on estimated life expectancy, with values representing the life expectancy of persons at the median in a specific population [18]. However, most life tables models do not take comorbidity into account. We argue that our alternative statistical model where we used age, CCI, and DCI collected at a population-based level is useful for estimates of remaining lifetime. By adding comorbidities based on prescriptions dispensed, which captures the outpatient populations comorbidity [3, 12], this increased the accuracy of predicting life expectancy. This makes our method particularly useful in older men with CCI = 0, since age and CCI alone do not appear to be sufficiently predictive of life expectancy. The validation performed in our study is designed for applications on a population-based level. To use the life expectancy presented in Fig. 2 as a clinical decision tool for individual patients, further validations of our methodology is required.

The need for information on life expectancy is pertinent for clinical guideline recommendations. For example, the European Association of Urology Guidelines Office recommends that men diagnosed with intermediate-risk prostate cancer should be treated with curative intent if the life expectancy is > 10 years [19, 20]. This is difficult to quantify as age and comorbidity in the context of life expectancy form a dynamic process. In addition, the quantification of life expectancy is potentially hampering recruitment to randomised clinical trials, as age and comorbidities are often a important components of eligibility criteria [21].

**Fig. 7:**
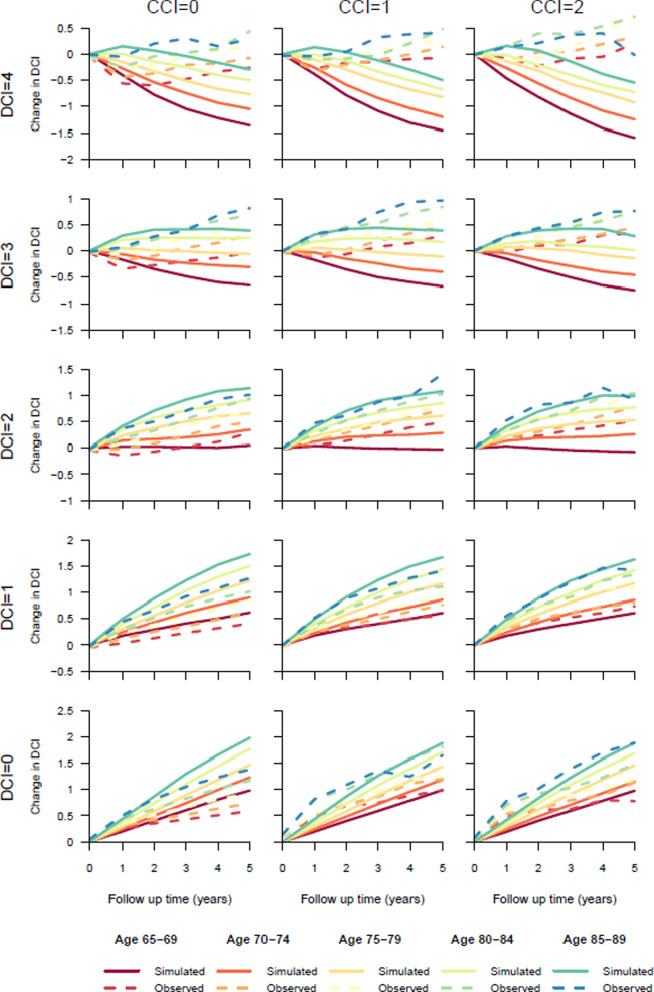
Estimated and observed mean change in DCI based on CCI and age

**Fig. 8:**
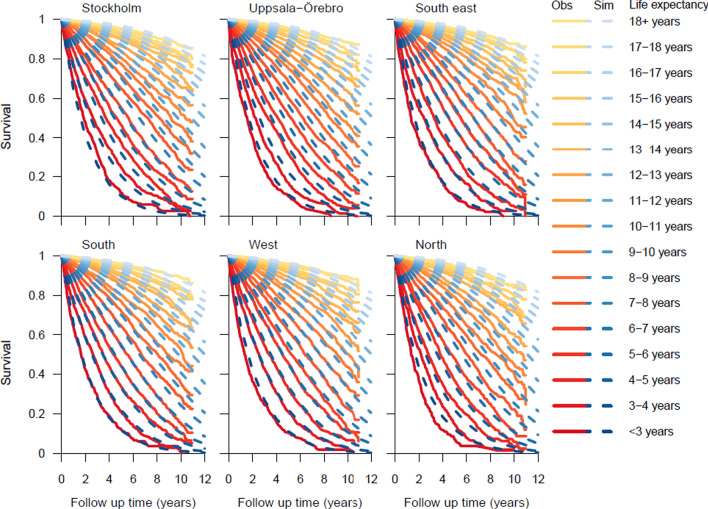
Cross validation of method to estimate life expectancy based on data from different regions in Sweden

**Fig. 9:**
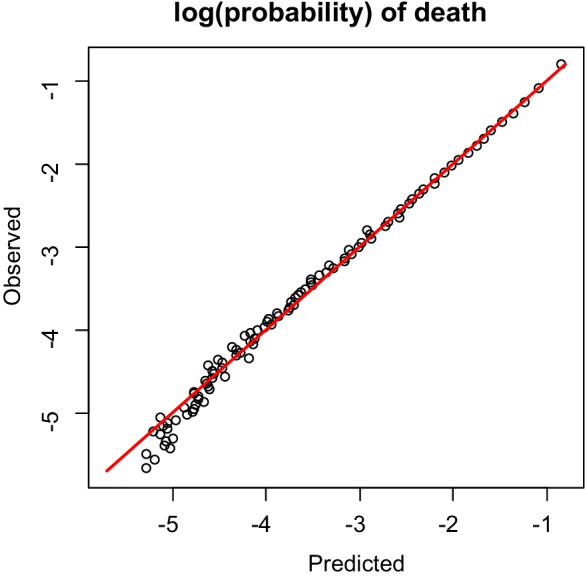
Calibration plot for the probability of death model

**Fig. 10:**
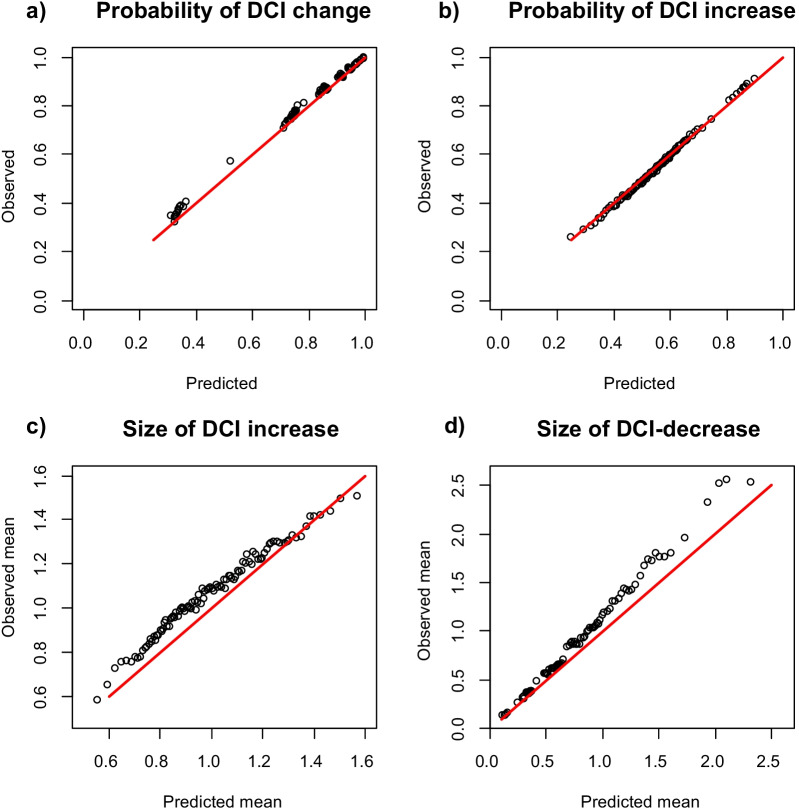
Calibration plots for the change in DCI models

**Fig. 11:**
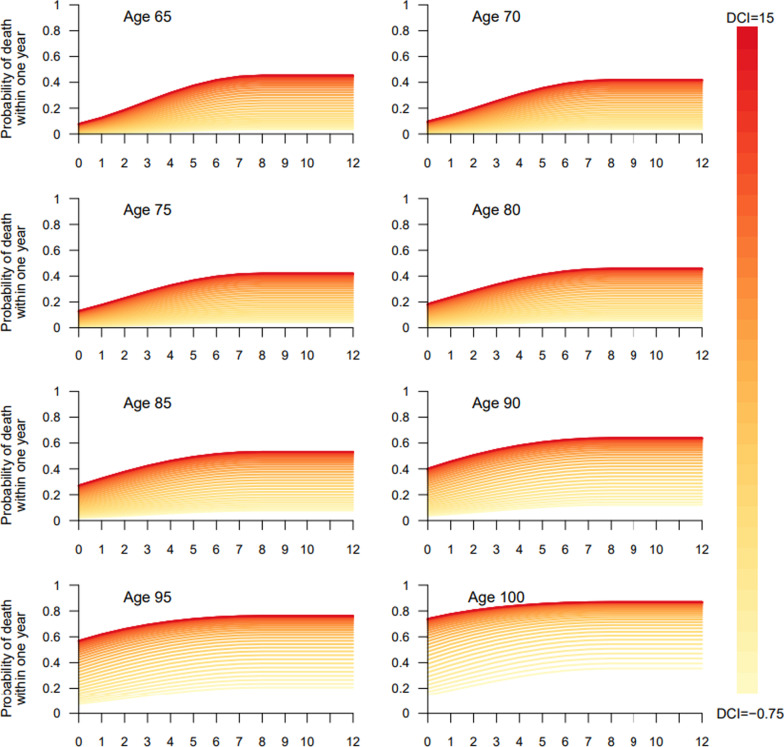
Modelled probability of death within 1 year based on age and levels of CCI, DCI

A limitation of our model is that it was developed for men. Whilst the estimates for females will be different, it is expected that the same basic methodology will be applicable. Another limitation of the current dataset is that prostate cancer was not a comorbidity at baseline. All men were free of prostate cancer at time of cohort entry. Men diagnosed with prostate cancer during follow-up were, however, considered in the dynamic CCI. Given that this was the case for the entire cohort and recongnising the consistent results of our external validity (Fig. 8), this does not affect the statistical properties of the model proposed in terms of estimating life expectancy. Furthermore, we only used information on current CCI/DCI levels—i.e. information about duration of past CCI/DCI levels was not considered. This makes the model easier to interpret, but also has some drawbacks. For example, a DCI of 2.0 that has not changed for 2 years prior to date of analysis is not likely to have the same impact on survival probability as a DCI of 2.0 that changed from an earlier level of 10. The latter man likely has a much higher risk of dying in the next year as frailty may affect drug prescriptions [22]. Moreover, it is unlikely for the DCI to decrease in two consecutive years—even though our model allows for this. This can be observed in Fig. 7 where the simulated curve allowed for consecutive drops in DCI and the observed curve rarely showed this. Our choice of modelling the size of DCI-changes using the Gamma distribution to underestimated the changes mildly. In men with long survival, the effects of repeated underestimations might accumulate and cause less accurate survival predictions. This might explain why the model was found to work less well for young men with very long life expectancy. Risk of death for these men was less likely to be associated with existing comorbidities or drug prescriptions but more likely with accidents or violence, hence errors in predicted DCI and CCI might accumulate over a long follow-up time.

Whilst our methods were accurate, they can only be applied to geographical regions where similar registries are available as it is key to capture the dynamic process of changes in comorbidities.

Future work to improve our proposed methods will involve calibrations based on proportion of deaths from injuries not related to CCI or DCI as well as inclusion of information about CCI and DCI changes prior to the current stage (i.e. allow for non-Markovian properties).

## Conclusion

Our proposed method provides a way of estimating life expectancy at the population level, whilst considering current comorbidity assessed by discharge diagnoses and data on filled prescriptions from nationwide population-based registries. In clinical practise, these estimates of life expectancy can be used to inform development of treatment guidelines and to improve inclusion criteria for RCTs.

## Data Availability

Data used for the current study can be retrieved by contacting hans.garmo@kcl.ac.uk. The steering groups of NPCR and PCBaSe welcome external collaborations. For more information please see www.npcr.se/in-english where registration forms, manuals, and annual reports from NPCR are found as well as a full list of publications from PCBaSe.

## Appendix

See Tables 1, 2, 3, 4 and Figs. 6, 7, 8, 9, 10 and 11.

**Table 1:** Variables used in the long dataset

Variable	Description	Values
Dead	Vital status	True/false (T/F)
Age	Age at start of time step	65, 66, …. 105
CCI	CCI level at start of time step	0, 1, 2 …
DCI	DCI level at start of time step	Continuous
QCS(x, y)	QCS = x^2 if x < y and y^2 for x ≥ y	Continuous
Any.CCI.change	CCI change during the time step	True/false (T/F)
Any.CCI.change6	CCI change of size 6 during the time step	True/false (T/F)
CCI.change	CCI change during time step	0, 1, 2 …
CCI.fct	CCI factorised	0/1/2/3/4+
CCI.fct.6p	CCI factorised	0/1/2+
Any.DCI.change	DCI change during the time step	True/false (T/F)
DCI.increase	DCI increased during the time step	True/false (T/F)
DCI.fct	DCI factorised	< 0/0/0–0.25/0.25–1/1–2/2–3/3–4/4–5/5–7/7+
DCI.change	DCI at end of time step minus DCI at start of time step	Continuous

**Table 2:** Models used for each time step of the proposed algorithm

Step in algorithm	Type of generalised linear model	Outcome	Model as specified in R-code
1	Logistic regression	Dead	Age + QCS(Age,100)*QCS(CCI,8) + QCS(Age,100)*QCS(DCI,14) + QCS(Age,100)*QCS(CCI,8)*QCS(DCI,14)
3	Logistic regression	Any.CCI.change	Age + DCI.fct + CCI.fct
3	Poisson regression	CCI.change	Age + DCI.fct + CCI.fct
3	Logistic regression	Any.CCI.change6	Age + DCI.fct + CCI.fct.6p + CCI
5	Logistic regression	Any.DCI.change	Age + dci.fct + cci.fct + Age*cci.fct + Age*dci.fct
5	Logistic regression (restricted to men with DCI-change)	DCI.increase	Age + dci.fct + cci.fct + Age*cci.fct + Age*dci.fct
6	Gamma regression (restricted to men with DCI-increase)	DCI.change	Age + dci.fct + cci.fct + Age*cci.fct + Age*dci.fct
7	Gamma regression (restricted to men with DCI-decrease)	-DCI.change	Age + dci.fct + cci.fct + Age*cci.fct + Age*dci.fct

**Table 3:** Years lost during ten first years of follow up according to observed and simulated survival, by groups of expected remaining lifetime

Expected remaining lifetime	Observed	Simulated	Difference
< 3 years	7.81	7.67	0.13
3–4 years	6.94	6.76	0.18
4–5 years	6.07	5.91	0.16
5–6 years	5.22	5.09	0.14
6–7 years	4.53	4.30	0.24
7–8 years	3.75	3.63	0.12
8–9 years	3.14	3.04	0.10
9–10 years	2.60	2.55	0.05
10–11 years	2.17	2.11	0.06
11–12 years	1.81	1.76	0.05
12–13 years	1.45	1.44	0.00
13–14 years	1.21	1.22	− 0.01
14–15 years	1.02	0.99	0.03
15–16 years	0.85	0.81	0.04
16–17 years	0.70	0.68	0.02
17–18 years	0.61	0.54	0.08
18+ years	0.50	0.44	0.06

**Table 4:** Years lost during ten first years of follow up by regions in Cross validation. Retrieved from observed and simulated survival, by groups of expected remaining lifetime

Expected remaining lifetime	Stockholm			Uppsala–Örebro			South east			South			West			North		
	Obs	Sim	Diff	Obs	Sim	Diff	Obs	Sim	Diff	Obs	Sim	Diff	Obs	Sim	Diff	Obs	Sim	Diff
< 3 years	7.52	7.78	− 0.26	7.96	7.68	0.28	7.90	7.71	0.18	7.66	7.66	0.00	7.92	7.71	0.20	8.03	7.63	0.40
3–4 years	6.62	6.90	− 0.28	7.16	6.76	0.40	6.95	6.83	0.13	6.89	6.80	0.09	6.86	6.79	0.08	7.35	6.77	0.58
4–5 years	5.76	6.06	− 0.30	6.19	5.86	0.33	6.13	5.94	0.20	5.90	5.97	− 0.07	6.04	5.92	0.12	6.54	5.89	0.64
5–6 years	5.04	5.20	− 0.16	5.45	5.01	0.44	5.08	5.12	− 0.04	5.19	5.17	0.02	5.11	5.10	0.01	5.67	5.09	0.58
6–7 years	4.44	4.43	0.01	4.60	4.26	0.35	4.37	4.34	0.03	4.42	4.39	0.03	4.57	4.35	0.22	4.96	4.33	0.63
7–8 years	3.49	3.74	− 0.25	3.92	3.61	0.31	3.75	3.69	0.07	3.60	3.73	− 0.13	3.79	3.67	0.12	4.07	3.66	0.41
8–9 years	2.95	3.13	− 0.17	3.27	2.98	0.29	3.14	3.06	0.08	2.97	3.12	− 0.15	3.15	3.09	0.06	3.36	3.03	0.34
9–10 years	2.44	2.66	− 0.22	2.68	2.49	0.19	2.57	2.58	− 0.02	2.53	2.61	− 0.07	2.58	2.58	0.00	2.83	2.51	0.31
10–11 years	2.12	2.20	− 0.08	2.35	2.08	0.27	2.06	2.16	− 0.10	2.10	2.19	− 0.09	2.13	2.11	0.01	2.35	2.07	0.29
11–12 years	1.76	1.82	− 0.06	1.89	1.73	0.16	1.67	1.76	− 0.09	1.69	1.80	− 0.11	1.74	1.78	− 0.04	1.98	1.71	0.27
12–13 years	1.46	1.50	− 0.04	1.57	1.46	0.11	1.43	1.48	− 0.05	1.43	1.50	− 0.07	1.43	1.47	− 0.05	1.67	1.41	0.26
13–14 years	1.17	1.26	− 0.09	1.25	1.24	0.01	1.25	1.25	0.00	1.14	1.26	− 0.12	1.23	1.25	− 0.02	1.21	1.20	0.02
14–15 years	1.01	1.02	− 0.01	1.05	1.00	0.05	1.02	1.01	0.01	1.03	1.02	0.01	0.97	1.02	− 0.05	1.08	0.97	0.10
15–16 years	0.85	0.85	0.01	0.90	0.85	0.05	0.85	0.85	0.00	0.79	0.85	− 0.06	0.79	0.85	− 0.06	0.93	0.83	0.10
16–17 years	0.75	0.69	0.06	0.70	0.67	0.03	0.66	0.69	− 0.03	0.71	0.70	0.01	0.70	0.69	0.01	0.80	0.67	0.13
17–18 years	0.64	0.54	0.10	0.58	0.54	0.04	0.61	0.55	0.06	0.59	0.57	0.02	0.52	0.55	− 0.03	0.67	0.54	0.13
18+ years	7.52	7.78	0.10	0.49	0.43	0.06	0.51	0.43	0.08	0.46	0.43	0.03	0.47	0.44	0.02	0.59	0.43	0.16
